# Foot-stiffening socks reduce the metabolic cost of walking

**DOI:** 10.1098/rspb.2026.1467

**Published:** 2026-11-04

**Authors:** Hui Tang, Owen Beck

**Affiliations:** Department of Kinesiology and Health Education, The University of Texas at Austin, Austin, TX 78712-1415, USA

**Keywords:** economy, biomechanics, arch, ankle, muscle, gearing, footwear, stiffness

## Abstract

Greater foot longitudinal bending stiffness theoretically facilitates bipedal walking by reducing whole-body metabolic rate, yet empirical evidence is elusive. Here, we tested this theory by studying human walking with and without foot-stiffening socks. These custom socks applied more pressure to the user's forefoot than commercially available socks (*p* < 0.001), which in turn increased medial longitudinal arch stiffness through cross-axis coupling (*p* = 0.034). Walking with foot-stiffening socks reduced whole-body metabolic rate 2% versus the use of commercial socks (*p* = 0.008). Biomechanically, stiffening participants' feet increased ankle gear ratio (peak: *p* = 0.017; average: *p* = 0.045) and reduced the ankle's average angular velocity during the stance-phase of walking (*p* = 0.018). In congruence, leg muscle fascicle shortening velocities decreased 15–20% (soleus: *p* = 0.023; medial gastrocnemius: *p* = 0.005), contributing to the 9–12% reduction in estimated leg active muscle volume (model_1_: *p* = 0.015; model_2_: *p* = 0.142). Therefore, foot-stiffening socks make walking less effortful by eliciting more economical leg muscle contractions, supporting the notion that the evolved stiffening of human feet facilitate terrestrial bipedal locomotion.

## 1. Introduction

Humans have stiffer feet than other primates [1–4]. Approximately 7–8 million years ago, the human lineage emerged from the last common ancestor with chimpanzees and bonobos [5]. Since this emergence, human ancestors developed distinct anatomical features that limit midfoot dorsiflexion during walking [4,6–8]. Features that stiffen the foot evolved concurrent to the increasing prevalence of terrestrial locomotion and may have been naturally selected to help facilitate bipedal walking [2,4,8–11].

Stiffer feet theoretically make walking less effortful. By resisting midfoot dorsiflexion, greater foot stiffness typically yields more mechanical work by the foot per step [12,13]. In turn, leg muscles require less mechanical work to sustain steady-state walking, which may reduce whole-body metabolic rate [14,15]. Additionally, stiffer feet may reduce metabolic rate by improving the economy and capacity of leg muscles to produce force and mechanical work. A stiffer foot propagates the centre of pressure further anteriorly during the stance-phase of walking [12,13]. This propagation increases ankle gear ratio, which is the ratio of the moment arm of the ground reaction force vector and ankle joint to the moment arm of the Achilles tendon and ankle joint [16]. A greater ankle gear ratio enables calf muscles to produce a unit of mechanical power using slower fascicle shortening velocities [12,13], which may reduce the volume of muscle activated during walking [17–19]. Thus, stiffer feet plausibly enable humans to walk with more efficient calf muscle contractions and/or greater calf muscle work production, both of which likely reduce metabolic rate during walking [20–22]. The notion that stiffer feet reduce the effort required to walk supports theories of human bipedalism [2,4,9–11,23,24] and informs interventions aimed to facilitate walking in modern-day humans [12,13,25].

Despite the notion that stiffer feet should reduce the metabolic rate during walking, no foot-stiffening intervention has realized this potential at ecologically relevant speeds. No study has reduced the metabolic rate of walking by stiffening the biological feet of an ape. Effectively increasing foot stiffness using shoes with stiffer midsoles does not reduce metabolic rate during walking for young or older adults under the speed at which humans transition to running [12,13,26–28]. Moreover, inhibiting plantar intrinsic muscles that actively stiffen the medial longitudinal arch does not affect metabolic rate during walking [29] nor does restricting medial longitudinal arch compression using shoe inserts [30]. The inability of foot(wear)-stiffening interventions to reduce metabolic rate during walking are unable to support pervasive theories of human bipedalism [2,4,9–11,23,24] or increase the walking endurance of modern-day humans [27,31,32].

Perhaps foot-stiffening interventions do not reduce metabolic rate during walking because they hinder other aspects of foot function. After resisting midfoot dorsiflexion for most of ground contact, the medial longitudinal arch plantarflexes through terminal stance [3,33]. Late stance also incurs metatarsophalangeal joint flexion, which affects medial longitudinal arch mechanics [7,34–36]. Restricting these late-stance movements reduces foot-ankle mechanical power and work production, in addition to eliciting more flexed lower-limb posture [12,13,30,33,36]. Because foot-stiffening interventions typically limit late-stance foot mobility [12,13,30,36,37], the metabolic benefits of walking with stiffer feet may be masked by uneconomical terminal stance-phase mechanics.

Here, we sought to determine whether stiffer feet make walking less effortful. We hypothesized that increasing foot stiffness would reduce whole-body metabolic rate during walking if late-stance foot mobility is unimpaired. To test this hypothesis, we created foot-stiffening socks (figure 1). These socks applied approximately 12 times more pressure to the user's forefoot than common commercially available socks, along a 3 cm wide area that encircles the metatarsal heads. By applying more pressure to the forefoot, foot-stiffening socks mitigate foot splay and increase the stiffness of the medial longitudinal arch and the overall foot through cross-axis coupling [4]. During walking, midfoot dorsiflexion occurs throughout the initial 80% of stance [3] and primarily depends on tarsometatarsal joint flexion. Owing to the (mis)alignment of the metatarsals, sagittal-plane flexion causes forefoot splay. By resisting forefoot splay, such as by adding textiles in-parallel to the foot, greater sagittal-plane moments are required to displace the metatarsals—increasing medial longitudinal arch and overall foot stiffness. Our previous study empirically demonstrated that restricting forefoot splay increases foot stiffness without altering metatarsophalangeal joint flexion, late-stance medial longitudinal arch plantarflexion or the mechanical work produced by the arch or ankle during running [38]. Hence, we designed socks to increase foot stiffness while maintaining late-stance foot mobility, enabling us to test our hypothesis by studying how foot-stiffening socks affect human metabolic rate during walking versus the use of compliant nylon foot coverings (control socks; figure 1).

**Figure 1.**
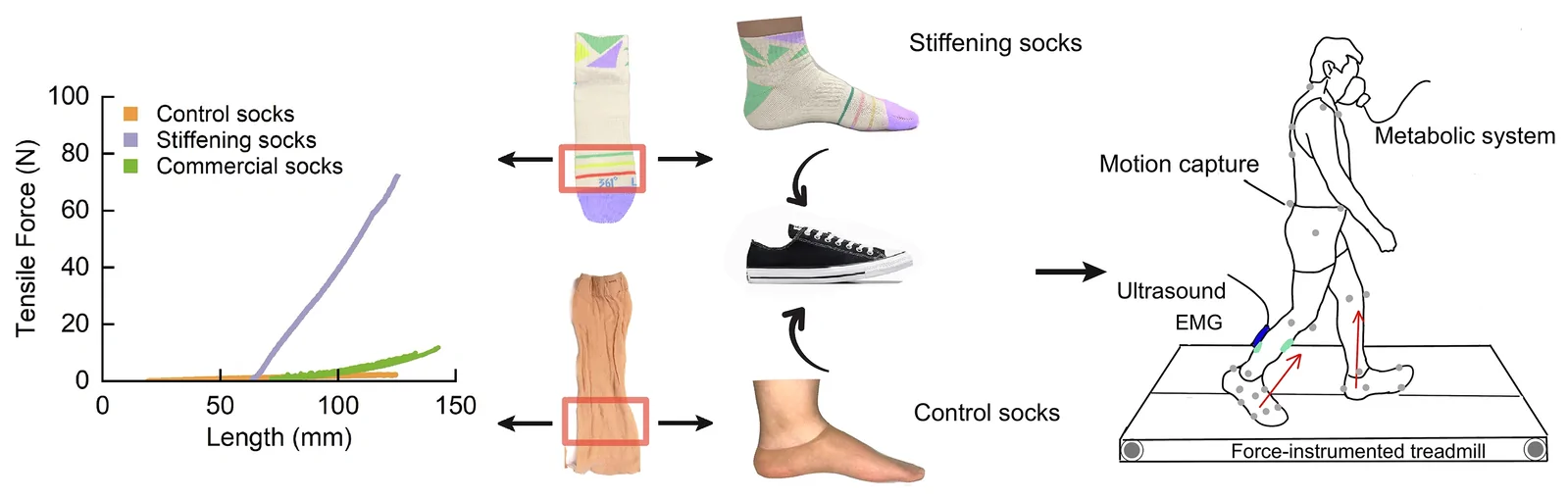
The testing of foot-stiffening socks. (Left) Tensile force versus length profiles of the control socks (orange), foot-stiffening socks (purple), as well as two representative commercially available socks (green) at the region that encircles the forefoot (denoted by the red rectangle). (Right) Twelve participants wore control socks and foot-stiffening socks during shod treadmill walking in a counterbalanced order (*n* = 6 ABBA, *n* = 6 BAAB). During treadmill walking, we recorded expired gas using a metabolic system, ground reaction forces via force-instrumented treadmill, body movement patterns using a motion capture system, leg muscle activation using surface electromyography (EMG) and leg muscle fascicle images via B-mode ultrasonography.

## 2. Results

Foot-stiffening socks mitigated forefoot splay and stiffened the medial longitudinal arch. Foot-stiffening socks applied approximately 24 times more pressure to the forefoot than control socks (*d* = 3.77, *p* < 0.001, 95% CI = 155, 178; figure 2A). In turn, foot-stiffening socks reduced standing forefoot splay (*d* = 0.48, *p* = 0.011, 95% CI = −0.5, −0.1) and elicited 10% narrower feet versus the use of control socks (*d* = 1.47, *p* < 0.001, 95% CI = −1.1, −0.7; figure 2B,C). Through cross-axis coupling, foot-stiffening socks increased medial longitudinal arch stiffness by 65% versus the use of control socks (*d* = 0.70, *p* = 0.003, 95% CI = 11.9, 67.2; figure 2D).

**Figure 2.**
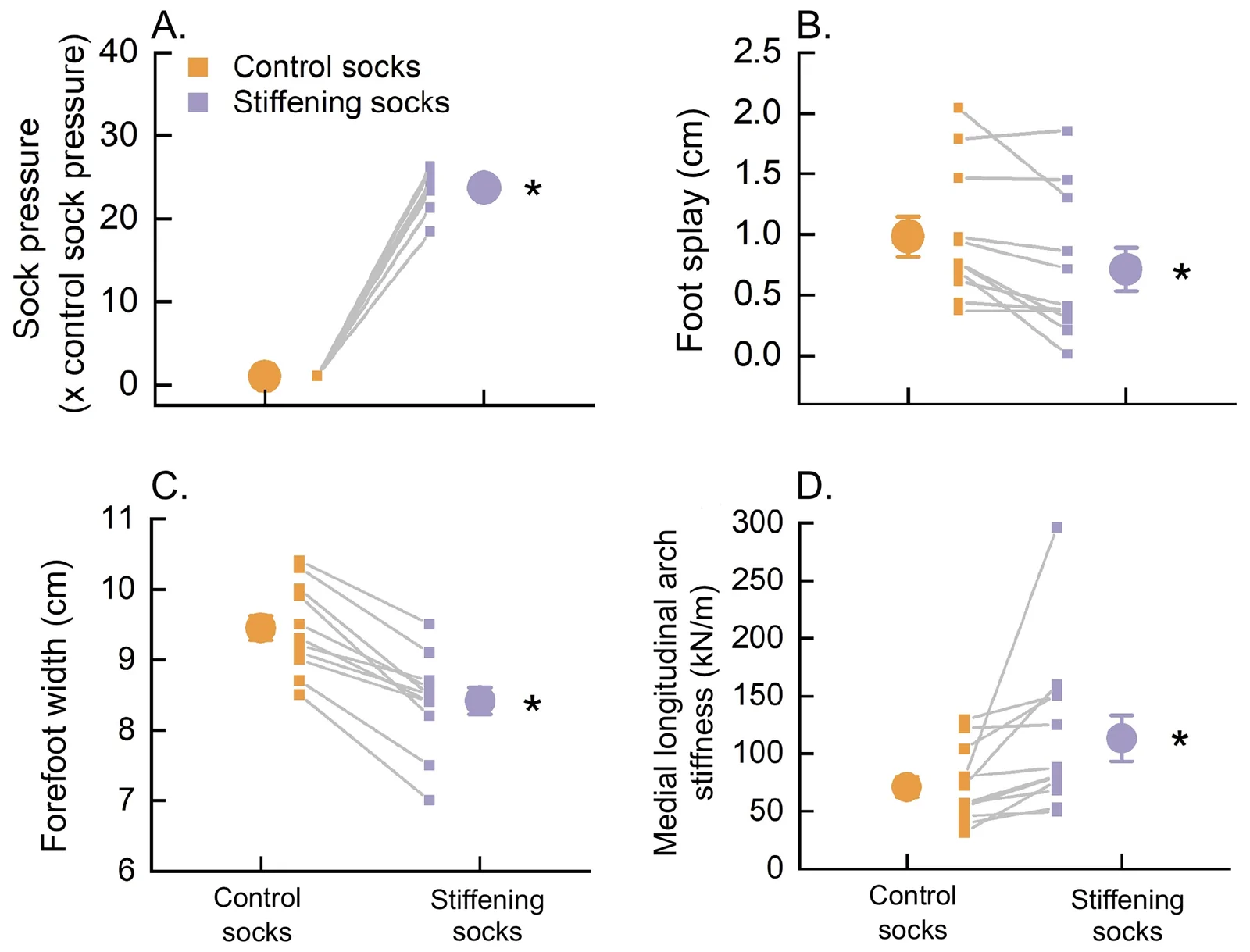
Foot-stiffening socks increased foot stiffness. Compared with control socks (orange). Foot-stiffening socks (purple) (A) applied more pressure along the forefoot, (B) eliciting less foot splay during sit-to-stand and (C) narrower feet during an unloaded condition. (D) Foot-stiffening socks increased medial longitudinal arch stiffness versus control socks. Circle symbols represent participant Avg ± s.e. and square symbols represent individual data. Asterisks indicate significance (*p* < α).

Foot-stiffening socks reduced user metabolic rate during walking. Walking in foot-stiffening socks reduced net metabolic power in 10 of 12 participants compared with walking in control socks (figure 3A,B). Across all participants, net metabolic power decreased 2% in foot-stiffening versus control socks (*d* = 0.93, *p* = 0.008, 95% CI = −0.11, −0.02; figure 3A,B).

**Figure 3.**
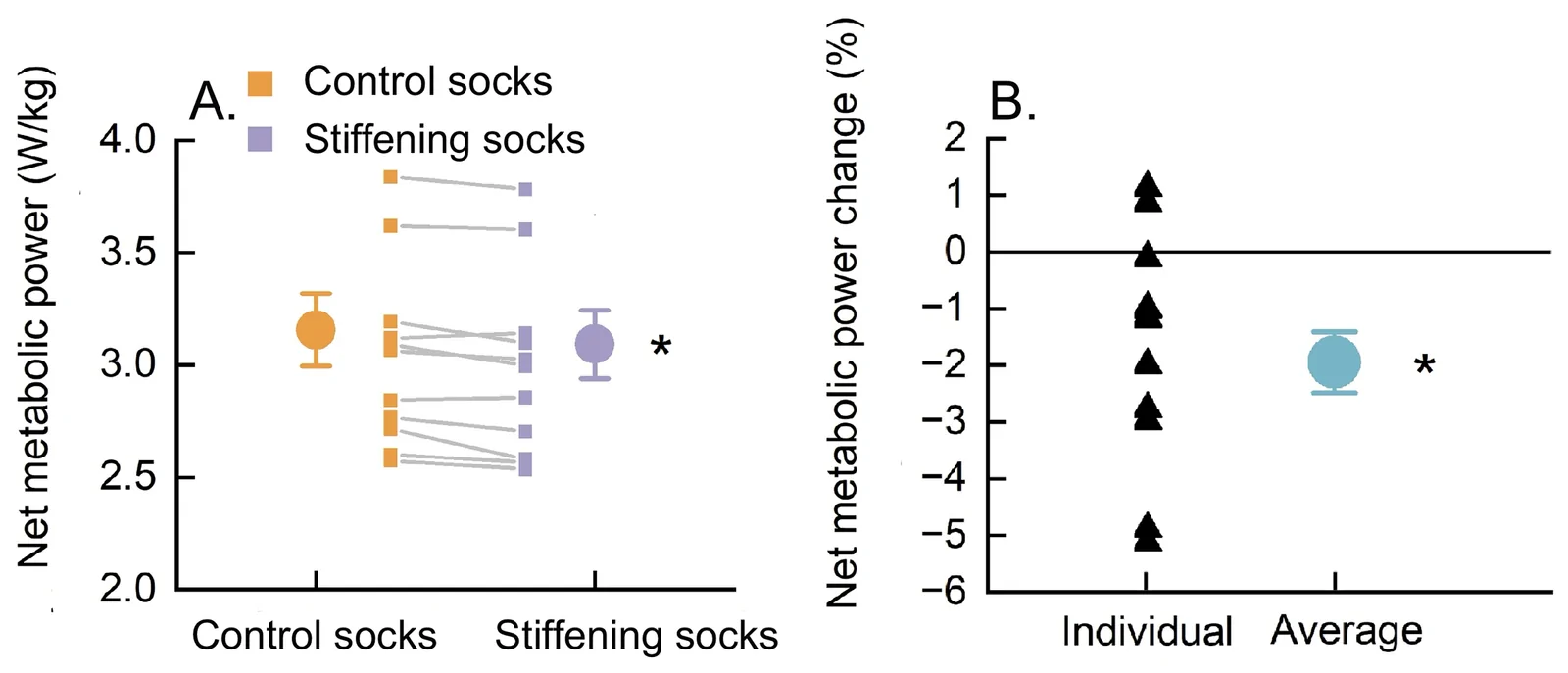
Foot-stiffening socks reduced whole-body net metabolic power during walking. Compared with walking with control socks (orange), foot-stiffening socks (purple) decreased whole-body net metabolic power. (A) Net metabolic power during walking versus footwear condition presented as participant Avg ± s.e. (circle symbols) and individual data (square symbols). (B) Percentage change in net metabolic power during walking in foot-stiffening socks versus compliant-control socks per individual (triangle symbols) and Avg ± s.e. (circle symbol). Asterisks indicate significance (*p* < α).

Foot-stiffening socks increased ankle gear ratio. Consistent with walking in stiffer shoes [12,13], foot-stiffening socks shifted the centre of pressure anteriorly during ground contact. Participant average (*d* = 0.68, *p* = 0.045, 95% CI = 0.004, 0.65) and peak (*d* = 0.51, *p* = 0.017, 95% CI = 0.07, 0.92) ankle gear ratios were greater during walking in foot-stiffening socks versus control socks (figure 4B), which increased stance average ankle moments 11% (*d* = 0.48, *p* = 0.045, 95% CI = 0.04, 11.18; figure 4C,D). Importantly, participants maintained nearly identical ankle mechanical power profiles (peak: *d* = 0.03, *p* = 0.925, 95% CI = −0.7, 0.7; average: *d* = 0.21, *p* = 0.229, 95% CI = −2.0, 7.5) and mechanical work output across sock conditions (*d* = 0.17, *p* = 0.357, 95% CI = −0.018, 0.045; figure 4G,H), necessitating a reduction in average ankle angular velocity using foot-stiffening socks (*d* = 0.50, *p* = 0.018, 95% CI = −3.9, −33.5; figure 4E,F).

**Figure 4.**
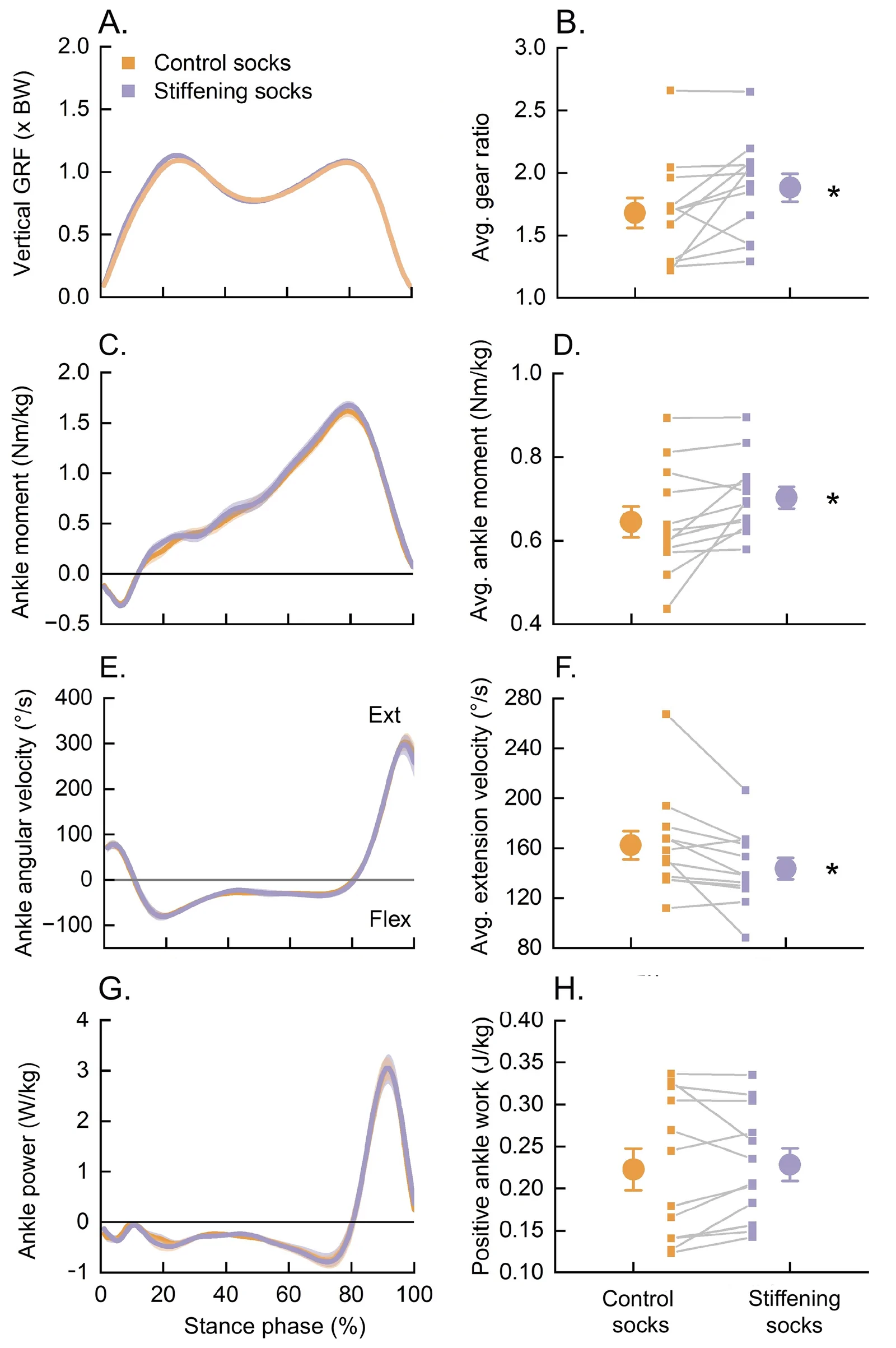
Foot-stiffening socks increased ankle gear ratio and did not affect ankle mechanical power or work production. (Left) Mechanical variables plotted over stance phase. (Right) Participant Avg ± s.e. (circle symbols) and individual participant data (square symbols) during walking in control socks (orange) and foot-stiffening socks (purple). Compared with walking with control socks, foot-stiffening socks (A) slightly increased peak vertical ground reaction forces (GRFs), in addition to (B) increasing the ankle's average gear ratio and (C, D) moments. Participants also walked with (E, F) reduced average ankle angular velocities in foot-stiffening socks. The increased ankle moments and reduced ankle angular velocities produced non-different mechanical power profiles across sock conditions. Participants walked with similar (G, H) ankle mechanical power and work output. Extension and flexion are abbreviated as Ext. and Flex., respectively. Asterisks indicate significance (*p* < α).

Foot-stiffening socks did not detectably limit late-stance foot mobility, alter spatiotemporal characteristics or affect proximal lower-limb joint mechanics. Both sock conditions exhibited non-different peak metatarsophalangeal flexion angles (*d* = 0.06, *p* = 0.681, 95% CI = −3.4, 2.3). Foot-stiffening socks did not increase ankle flexion at terminal ground contact (*d* = 0.05, *p* = 0.859, 95% CI = −2.8, 3.3; supplementary material, figure S1), which is apparent when walking with stiffer midsoles [12,13] and related to limited midfoot mobility [33]. Furthermore, participants displayed non-different ground contact time (*d* = 0.20, *p* = 0.210, 95% CI = −0.01, 0.03), stride frequency (*d* = 0.13, *p* = 0.509, 95% CI = −0.03, 0.2) and duty factor (*d* = 0.15, *p* = 0.224, 95% CI = −0.003, 0.008) across sock conditions. Both sock conditions yielded similar hip and knee moments and mechanical power output (supplementary material, figures S2 and S3). Because foot-stiffening socks affect ankle mechanics but not hip or knee mechanics, and that intrinsic foot muscles do not detectably alter whole-body metabolic rate *per se* owing to their small volume [29], the more economical walking in foot-stiffening versus control socks is likely due to differences in ankle muscles.

Foot-stiffening socks reduced ankle extensor muscle fascicle shortening velocity. Owing to the greater ankle gear ratio during walking with foot-stiffening socks compared with control socks, ankle extensor muscles produced more force while contracting at slower shortening velocities. On average, soleus muscle fascicles produced 15% more force during walking with foot-stiffening socks versus control socks (*d* = 0.50, *p* = 0.045, 95% CI = 0.03, 2.12; figure 5A,B; supplementary material, figure S4). Consistent with ankle kinetics, sock condition did not affect soleus or medial gastrocnemius average fascicle mechanical power (soleus: *d* = 0.20, *p* = 0.40, 95% CI = −0.09, 0.02; medial gastrocnemius: *d* = 0.35, *p* = 0.090, 95% CI = −0.025, 0.002) or work output (soleus: *d* = 0.26, *p* = 0.40, 95% CI = −0.08, 0.01; medial gastrocnemius: *d* = 0.35, *p* = 0.090, 95% CI = −0.015, 0.001). Thus, walking with foot-stiffening socks reduced average soleus and medial gastrocnemius fascicle contractile velocities by 20% and 15%, respectively (soleus: *d* = 0.69, *p* = 0.023, 95% CI = −2.33, −0.13; medial gastrocnemius: *d* = 0.52, *p* = 0.005, 95% CI = −2.89, −0.34; figure 5E,F; supplementary material, figure S4). Sock condition did not affect peak or average soleus or medial gastrocnemius fascicle pennation angle or operating length (soleus: *d* = 0.07, *p* = 0.826, 95% CI = −1.0, 0.8; medial gastrocnemius: *d* = 0.02, *p* = 0.763, 95% CI = −1.1, 0.9; figure 5C,D; supplementary material, figure S4). Therefore, walking with foot-stiffening socks increased ankle extensor muscle fascicle force production and reduced the corresponding muscle contractile velocities.

**Figure 5.**
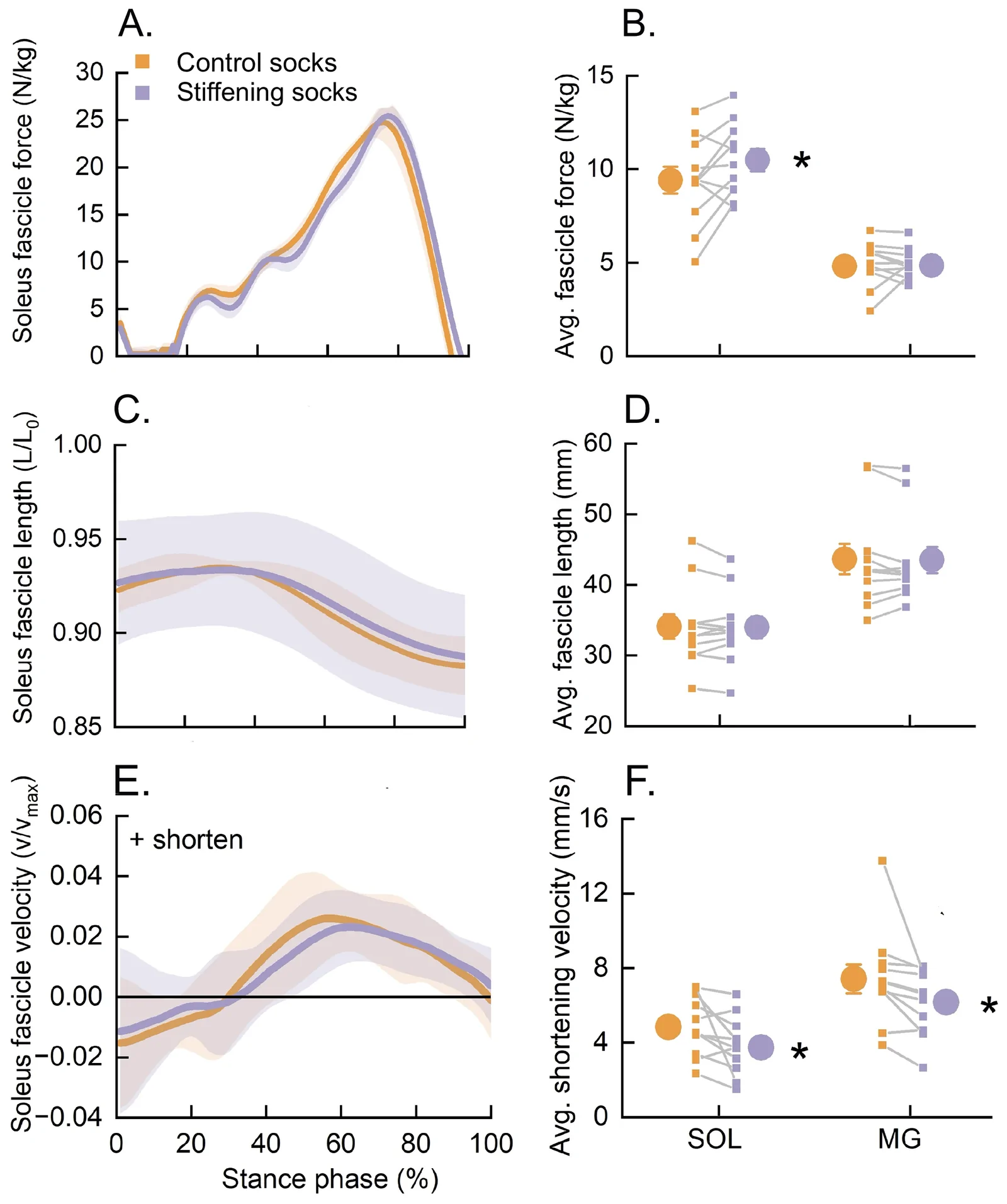
Foot-stiffening socks increased ankle extensor muscle fascicle force production and decreased the concurrent shortening velocity. (Left) Soleus (SOL) mechanical variables plotted over the stance phase. (Right) Soleus and medial gastrocnemius (MG) participant Avg ± s.e. (circle symbols) plus individual data (square symbols) during walking in control socks (orange) and foot-stiffening socks (purple). Compared with walking with control socks, foot-stiffening socks (A, B) increased average soleus muscle fascicle force, (C, D) did not alter soleus or medial gastrocnemius length and (E, F) decreased average soleus and medial gastrocnemius shortening velocity. Asterisks indicate significance (*p* < α).

Foot-stiffening socks reduced modelled ankle extensor active muscle volume. To explain how foot-stiffening socks reduced whole-body metabolic rate, we estimated ankle extensor active muscle volume using two distinct methods [19]. Active muscle volume relates to whole-body metabolic power because it is a proxy for the number of sarcomeres actively utilizing adenosine triphosphate and calcium pumping. First, we estimated soleus and medial gastrocnemius active muscle volume by combining ankle-joint and muscle-fascicle analyses with multiple model-based assumptions of triceps surae physiology (see Material and methods). Using this method, soleus plus medial gastrocnemius average active muscle volume rate decreased 12% using foot-stiffening versus control socks (*d* = 0.33, *p* = 0.015, 95% CI = −1.0, −0.1; figure 6A,B; supplementary material, figure S5). Second, we estimated soleus plus medial gastrocnemius active muscle volume using surface electromyography (EMG) signals and anatomical assumptions. Using this alternative method, walking with foot-stiffening socks numerically, but not statistically, reduced average soleus plus medial gastrocnemius active muscle volume rate 9% compared with control socks (*d* = 0.13, *p* = 0.142, 95% CI = −0.94, 0.01; figure 6C,D). Based on the estimate that ankle extensors constitute 27% of whole-body metabolism during walking [39], a 9–12% reduction in triceps surae active muscle volume predicts a 2–3% decrease in whole-body metabolic power, which aligns with our observed 2% decrease in net metabolic power.

**Figure 6.**
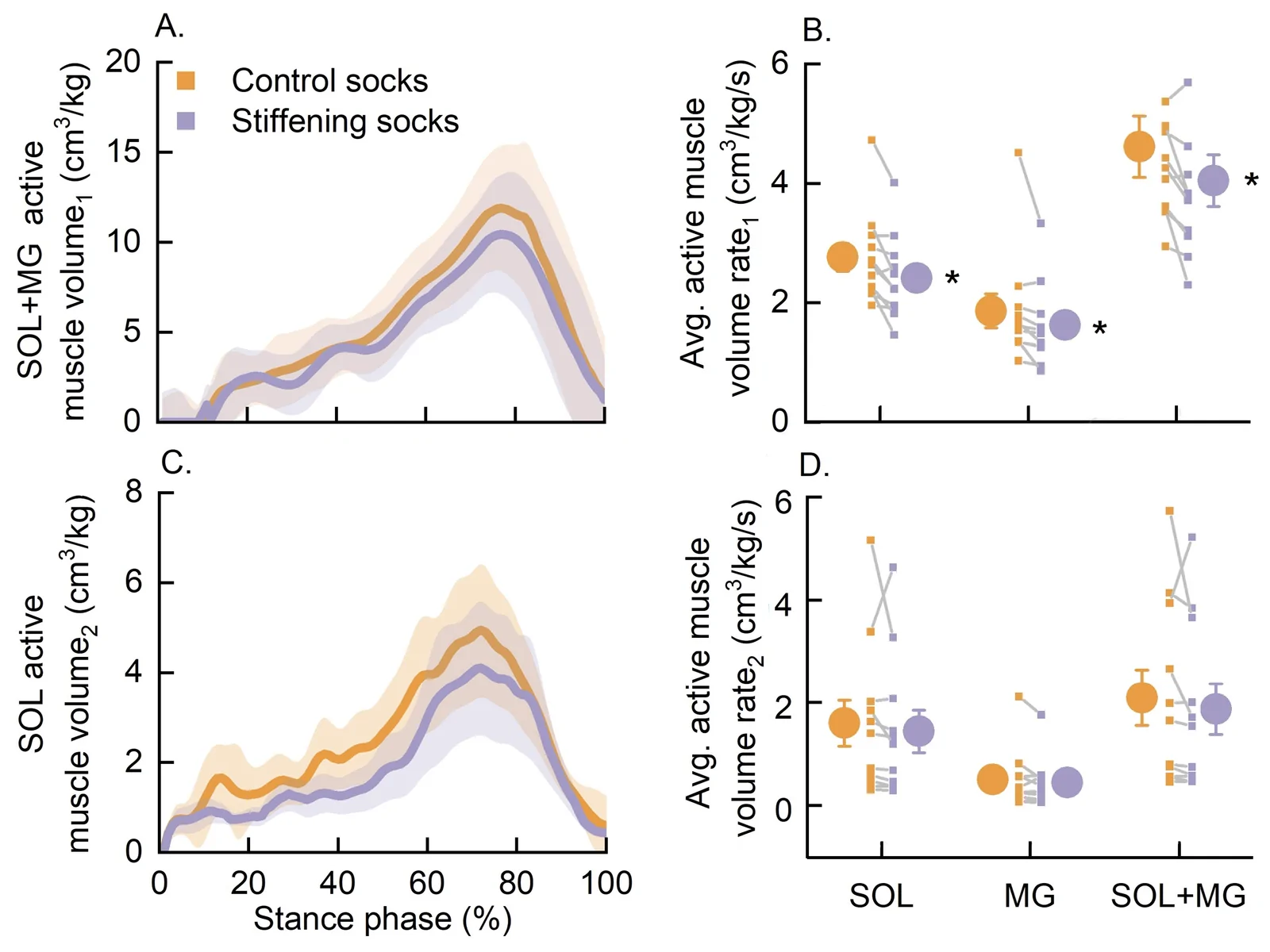
Foot-stiffening socks decreased modelled ankle extensor active muscle volume. (A) Modelled soleus (SOL) plus medial gastrocnemius (MG) active muscle volume over the stance phase using method 1. (C) Modelled SOL active muscle volume over the stance phase using method 2. (B, D) Active muscle volume rate of soleus, medial gastrocnemius and the summed total of both muscles for participant Avg ± s.e. (circle symbols) plus individual data (square symbols) during walking in control socks (orange) and foot-stiffening socks (purple) using the respective methods. Both methods of computing active muscle volume used numerous anatomical and physiological assumptions (see Material and methods); active muscle volume_1_ used muscle fascicle mechanics, whereas active muscle volume_2_ used surface electromyography (EMG) signals. Asterisks indicate significance (*p* < α).

## 3. Discussion

Foot-stiffening socks reduced whole-body metabolic rate during walking. These socks mitigated forefoot splay and stiffened the medial longitudinal arch without apparently limiting late-stance foot mobility. Supporting our hypothesis, walking with stiffer feet reduced user metabolic rate by eliciting more economical ankle extensor muscle contractions. These empirical findings support the notion that the evolved stiffening of human-ancestor feet promoted terrestrial bipedal locomotion [2,4,9–11,23,24,40]. In modern-day humans, foot-stiffening socks accomplished a goal yet to be achieved by shoes—reduce whole-body metabolic rate during walking at common speeds [12,13,26,27]. Therefore, by applying pressure to specific regions of the body, well-designed clothing offers a practical way to make walking less effortful throughout everyday life.

Foot-stiffening socks reduced whole-body metabolic rate by eliciting more economical ankle muscle contractions, not by reducing muscle mechanical work. Researchers conventionally surmise that stiffening human feet facilitate walking via two mechanisms: reduced leg muscle mechanical work and/or more economical leg muscle force production [13,30,41–45]. Steady-state walking requires the body to produce the same net mechanical work per step [46,47], and walking with stiffer feet reduces the mechanical energy stored and consequently dissipated by viscoelastic tissues per unit force [48,49]. Thus walking with stiffer feet is thought to reduce net mechanical work production from metabolically active muscles [50,51], but this potential benefit is commonly counteracted by greater forces acting on stiffer feet owing to decreased effective mechanical advantage [38,52]. Experimentally, we did not detect a difference in participant leg muscle fascicle or lower-limb joint mechanical work across footwear conditions, and similarly mitigating forefoot splay during running does not affect ankle or medial longitudinal arch net or positive mechanical work [38]. Current human-subject data does not support the notion that increasing foot stiffness sizably contributes to more economical bipedal terrestrial locomotion by reducing leg muscle mechanical work output. Rather, walking with increased foot stiffness likely reduces the metabolic rate of active leg muscles. Mechanistic research is warranted to deduce the source of the more economical muscle contractions as operating slower contractile velocities reduces active muscle volume to produce force but it also decreases muscle entropy efficiency [53].

Regardless of the mechanism, this study supports the notion that stiffer feet facilitate bipedal terrestrial locomotion. Since the human lineage emerged from the last common ancestor with chimpanzees and bonobos, hominins developed distinct anatomical features that increased foot stiffness [1,3]. Because these anatomical features evolved as hominins transitioned to more frequent bipedal walking [2,4,8–11,54], researchers reason that the stiffening of hominin feet facilitated bipedal walking [2,4,9–11,23,24,40], despite no supporting within-subject walking evidence. Here, our empirical findings support the claim that greater foot stiffness may have evolved to elicit more economical walking. And combined with the findings of human running experiments [38], anatomical features that stiffen the foot may have been naturally selected to facilitate bipedal walking and/or running.

Foot-stiffening socks present a low-cost, feasible way to improve human walking ability. By reducing whole-body metabolic rate 2%, foot-stiffening socks reduce the walking effort similar to doffing 2 kg from the waist [55], which theoretically increases walking endurance [24], reduces fatiguability [56] and may help lower barriers that limit participation in physical activity [57]. Due to these potential benefits of economical walking, researchers are constantly trying to develop wearable technology that reduce the effort required to walk (e.g. [58,59]). Today, rigid shoes are unable to reduce metabolic rate during walking at ecologically valid speeds [12,13,26,27] and exoskeletons are relatively expensive and difficult to use throughout daily life [56], despite their greater metabolic benefits [58,60]. Thus, we present foot-stiffening socks as a cheap and readily adoptable technology capable of reducing metabolic rate during walking, and perhaps running [38].

## 4. Material and methods

### (a) Participants

Twelve apparently healthy young adults participated (5 females, age: 28 ± 5 yrs; height: 1.76 ± 0.08 m; mass: 81.2 ± 12.8 kg, Avg ± s.d.). Before participation, everyone provided informed written consent as per The University of Texas at Austin's ethics committee approved protocol (approval number: STUDY00006562).

### (b) Foot-stiffening socks

We developed foot-stiffening socks using a blend of synthetic yarn (47% polyester and polyamide, 44% cotton and 9% elastane; LEIERFU Automatic Socks Knitting Machine, China). At a 30 mm wide region that incircles the forefoot, we tightly knitted 3 × 1 mm bands (supplementary material, figure S6). The tightly knitted bands were separated by 13.5 mm, yielding a total ‘forefoot region’ that is 30 mm wide.

We assessed foot-stiffening socks using a materials testing machine. We clamped the medial and lateral edges of the forefoot region in intact socks and performed uniaxial tensile testing (Instron, Norwood, MA, USA). Because sock height is minuscule while clamped, length measured by the materials testing machine equalled approximately half of the forefoot region's circumference. We stretched the forefoot region to the length that equalled half of the largest participant forefoot circumference three consecutive times. For each cycle, we applied a 1 N preload, stretched each sock at 8 mm s^−1^ and recorded data at 100 Hz. We performed the same testing using control socks, as well as standard cotton and diabetes socks for context (figure 1).

### (c) Foot mechanics

We measured participant foot mechanics in foot-stiffening socks, control socks and barefoot. After placing reflective markers over bony landmarks on the participant's dominant foot [61], we captured marker trajectories via infrared motion capture cameras at 100 Hz (Vicon, Oxford, UK) and ground reaction forces at 2000 Hz (Motek, Amsterdam, The Netherlands).

We quantified participant forefoot kinematics in each sock condition. We calculated foot splay as the change in distance between the first and fifth metatarsal head markers projected to the base of the foot when participants transitioned from sitting with their feet flat on the ground to quiet standing [38,62]. The ground reaction force amplitude was non-different between sock conditions during sitting (Average ± s.e.: control socks: 4.2% ± 3.8%; foot-stiffening socks: 4.4% ± 4.0% of body weight; *d* = 0.07, *p* = 0.744, 95% CI = −1.6, 2.1) or standing (control socks: 49.8% ± 2.0%; foot-stiffening socks: 50.2% ± 2.0% of body weight; *d* = 0.22, *p* = 0.727, 95% CI = −2.3, 3.2). We calculated the medial longitudinal arch stiffness for each sock condition during standing using the three-point bending model [42,62]. The compressive force acting on the medial longitudinal arch equalled the sum of Achilles tendon force and vertical ground reaction force, which is the magnitude of the ankle joint reaction force. To calculate stiffness, we took the quotient of the medial longitudinal arch's change in compressive force and respective change in navicular height displacement (medial longitudinal arch height) with respect to the foot's plantar surface plane from seated to standing [38,42,62].

We calculated forefoot pressure with foot-stiffening and control socks using Laplace's Law (equation (4.1)),

(4.1) $$P=\frac{T}{r_{1}},$$

where ‘P’ is pressure (N m^−2^), ‘T’ is tension per region width (N m^−1^) and ‘$r_{1}$’ is radius of the forefoot's curvature (m). We calculated T as half the tensile force recorded by the materials testing machine when sock length equalled half of the respective participant's forefoot circumference divided by forefoot region width (0.03 m; equations (4.2) and (4.3)). We approximated foot radius as participant forefoot circumference divided by 2π. We normalized forefoot pressure to that exerted by the control sock pressure (pressure in mmHg reported in supplementary material).

Materials testing yielded the following best fit linear relationships per sock condition:

(4.2)Control sock force (N)=0.02×sock length (mm)−0.3;R2=0.99(4.3)Footstiffeningsockforce(N)=1.19×socklength(mm)−77.5;R2=0.99.

Our methods probably overestimated pressure along the forefoot owing to unstretched sock material under the materials testing clamps across all conditions. Thus, we interpreted foot-stiffening sock pressure as a multiple of that measured from the commercially available sock used for comparison.

### (d) Experimental protocol

After measuring participant foot mechanics, we tested participants during shod treadmill walking in foot-stiffening socks and control socks. Participants completed five trials of 5 min walking at 1.25 m s^−1^ on the split-belt force-instrumented treadmill (Motek, Amsterdam, The Netherlands) with at least 5 min of rest between trials. The first trial served as treadmill familiarization where the participants walked in their personal footwear. For the subsequent trials, participants walked with shoes provided by the lab (Converse, Boston, MA, USA) and foot-stiffening socks or control socks in a counterbalanced order (*n* = 6 ABBA, *n* = 6 BAAB). The testing shoes had a flat sole *sans* arch support. During each walking trial, we collected participant metabolic and neuromechanics data.

### (e) Metabolic energy expenditure

We asked participants to arrive in the lab at least 3 h post-prandial to mitigate the influence of recently digested foodstuffs on metabolism. We used an indirect calorimetry system (TrueOne 2400, ParvoMedic, Sandy, UT, USA) to record participant rates of oxygen uptake (Vȯ_2_) and carbon dioxide production (Vċo_2_) throughout each walking trial. Using a standard equation [63], we calculated average metabolic power over the last 2 min during each walking trial. Next, we subtracted metabolic power during standing from each participant's walking trial and divided by mass to yield mass-specific net metabolic power (W kg^−1^) per walking trial.

### (f) Walking mechanics

We assessed participant walking mechanics during each trial using a force-measuring treadmill and a motion capture system (Vicon, Oxford, UK). Before walking, we placed 50 reflective markers on the participant's anatomical bony landmarks following IOR full body and foot models [61,64]. Using an 11-camera motion capture system, we collected marker trajectories (100 Hz) as participants walked on a split-belt force-measuring treadmill that sampled ground reaction forces (GRFs) at 2000 Hz. We filtered marker trajectories and GRFs using a fourth-order low-pass Butterworth filter with cutoff frequencies of 6 and 12 Hz, respectively.

We analysed the filtered data using Visual3D (HAS-Motion, Inc., Germantown, MD, USA) and custom MATLAB scripts (MathWorks, Natick, MA, USA). We implemented a 50 N vertical GRF threshold to identify the respective limb's stance and swing duration. We computed limb contact time, step frequency and duty factor. We calculated peak and average sagittal-plane ankle, knee and hip joint angles, moments, mechanical power and work during stance. After measuring each participant's Achillies tendon moment arm at a 90° ankle angle, we incorporated participant-specific scaling to calculate instantaneous moment arm using a second-order polynomial regression informed by magnetic resonance imaging data [13,65]. We calculated sagittal plane instantaneous ankle gear ratio throughout stance as the ratio of ground reaction force moment arm about the ankle joint centre to the Achilles tendon moment arm about the ankle joint centre. For each participant, we averaged whole-body and joint mechanics data from 10 strides per walking trial (20 steps per condition).

### (g) Muscle fascicle mechanics

During each walking trial, we also recorded soleus and medial gastrocnemius muscle fascicle length and pennation angles via a B-mode ultrasound probe that was secured to the participant's leg (53 Hz, Telemed, Vilnius, Lituania). We used a semi-automated tracking software to measure soleus and medial gastrocnemius fascicle lengths and pennation angles during walking [66]. When tracking results were unsatisfactory, we implemented manual corrections. We averaged soleus and medial gastrocnemius fascicle length and pennation angles across six steps during each walking trial (12 steps per condition). We computed muscle fascicle velocity as the derivative of fascicle length with respect to time.

We calculated soleus and medial gastrocnemius fascicle force during walking. To do so, we calculated ankle extensor muscle-tendon force (*F*_MTU_) by dividing net ankle moment ($M_{\text{ankle}}$) by the corresponding moment arm of the Achilles tendon ($r_{2}$; equation (4.4)). Muscle fascicle force (*F*_M_) for the soleus and medial gastrocnemius were computed as *F*_MTU_ multiplied by their respective cross-sectional area (CSA_m_) with respect to the total ankle extensor muscle CSA (CSA_tot_) [67]. Next, we divided muscle fascicle force by cosine of the respective muscle fascicle's pennation angle (*θ*_p_; equation (4.5)). Because soleus muscle fibres produce force along the ascending limb of their force-length profile [68], we deemed that there is no contributing passive force from ankle extensor muscles during walking

(4.4)FMTU=Mankler2(4.5)FM=FMTUcosθp⋅CSAmCSAtot.

We estimated soleus and medial gastrocnemius fascicle relative operating length and velocity during walking. To compute relative muscle fascicle operating length (L/L_0_), we divided instantaneous muscle fascicle length (L) by the assumed optimal length for active force production (L_0_). We assumed that soleus and medial gastrocnemius fascicles are at 93.8% of their optimal length for active force production during their maximal active length during the walking stance phase in the control sock condition [68]. To compute relative muscle fascicle velocity (V/V_max_), we divided instantaneous muscle fascicle velocity (V) by the assumed maximum shortening velocity (V_max_). Soleus and medial gastrocnemius comprise a mixture of muscle fibre ‘types’ (soleus: 81% type 1 and 19% type 2 fibres; medial gastrocnemius: 50% type 1 and type 2 fibres [69,70]). Type 1 and type 2 muscle fibres have a reported v_max_ at body temperature of 4.4 and 16.8 L_0_ s^−1^, respectively [71]. By scaling relative v_max_ estimates by the respective composition of each muscle, we calculated soleus and medial gastrocnemius fascicle v_max_ as 6.77 and 10.6 L_0_ s^−1^, respectively. Finally, we multiplied the respective muscle fascicle maximum shortening velocities (L_0_ s^−1^) by the respective optimal length for active force production (mm) to compute absolute maximum shortening velocity (mm s^−1^).

### (h) Muscle activity

We quantified the muscle activity of the medial gastrocnemius, soleus and the tibialis anterior using surface wireless electromyography on the left leg (Delsys Inc., Natick, MA, USA). Before data collection, we shaved and cleaned the participant's skin and adhered a sensor superficial to the respective muscle in the orientation of the underlying muscle fascicle. To enable normalization, maximal voluntary contractions (MVCs) were collected for each muscle. Participants performed three 5 s MVCs against manual resistance, with the highest peak EMG amplitude from any trial serving as the maximum value (100% MVC). Raw EMG signals were bandpass-filtered using a fourth-order Butterworth filter (20–400 Hz), full-wave rectified and low-pass filtered (fourth-order Butterworth, 6 Hz cutoff) to create a linear envelope. For each condition, root-mean-square (RMS) EMG was calculated over a 50 ms moving window and averaged across 20 steps, then normalized to %MVC to quantify average muscle activation amplitude (supplementary material, figure S7).

### (i) Muscle modelling and active volume estimation

We estimated soleus and medial gastrocnemius active muscle volume during walking using two complementary approaches. First, we computed active muscle volume ($V_{\text{act},1}$) using the respective muscle's fascicle force (*F*_m_, equation (4.5)) and optimal length for active force production (L_0_), along with an assumed muscle stress (σ = 20 N cm^−2^) [72] and muscle-force potential based on Hill-type force-length (FL) and force-velocity (FV) relationships [73–76] (equation (4.6))

(4.6) $$V_{\text{act},1}=\frac{F_{m}\cdot l_{0}}{\sigma \cdot FL\cdot FV}$$

We also computed active muscle volume ($V_{\text{act},2}$) using relative muscle activation (%MVC from surface EMG signals) and literature-reported soleus and medial gastrocnemius muscle volume from MRI data [77] (equation (4.7))

(4.7) $$V_{\text{act},2}=\text{act}\cdot V_{tot}$$

We divided each active muscle volume estimate by stride time to obtain the rate of active muscle volume (equation (4.8)), matching the time scale of metabolic measurements

(4.8) $${\dot{V}}_{\text{act}}=\frac{V_{\text{act}}}{t_{stride}}.$$

### (j) Statistics

To test our hypothesis, we designated net metabolic power as the primary outcome variable. All other footwear and neuromechanical variables were secondary measures. Secondary measures include foot mechanics (sock pressure, foot splay, foot width and medial longitudinal arch stiffness); spatiotemporal kinematics (ground contact time, stride frequency, duty factor); peak metatarsophalangeal joint angle; ankle mechanics (peak and average ankle gear ratio, angular velocity, joint angle and terminal ground contact, moment, power, work rate), soleus and medial gastrocnemius fascicle mechanics (peak and average fascicle force, length, velocity and pennation angle), as well as soleus and medial gastrocnemius active muscle volume. We tested the normality of the within-participant paired differences between control socks and foot-stiffening socks using the Shapiro–Wilk test. When the paired differences were consistent with a normal distribution, we performed a two-tailed paired-samples *t*-test. When normality was rejected, we performed a Wilcoxon signed-rank test. We set the significance threshold as α = 0.05 and conducted statistical analyses using RStudio (v4.3.2; Posit Inc., MA, USA). We reported Cohen's *d* effect sizes [78], and a mean difference 95% confidence intervals for the primary and key secondary variables.

## Supplementary Material



## Data Availability

All data and code needed to evaluate and reproduce the results are present in the main text, supplementary material files and a figshare repository [79].
