# Supplementary material for: Foot-stiffening socks reduce the metabolic cost of walking
Source: Proc Biol Sci. 2026 Nov 4;293(2082):20261467. doi: 10.1098/rspb.2026.1467 (PMC13730810; doi:10.1098/rspb.2026.1467)
Supplement: Supplementary file 1 [file rspb.2026.1467.zip › rspb.2026.1467/rspb20261467_si_002.docx]

**README**

Study: Foot-stiffening socks reduce the metabolic cost of walking.

For additional MATLAB & R codes, please refer to <https://doi.org/10.6084/m9.figshare.31412081>

File: SockWalk_SummaryData_0530.xlsx

Tab: Anthropometrical Data

A. Participant (number)
B. Sex (female or male)
C. Age (years)
D. Standing height (m)
E. Body mass (kg)
F. Shoe size (US women (F) /men (M))
G. Achilles tendon moment arm (cm)

Tab: Material Testing

A-C. Nylon sock materials testing

- B. Sock length (mm)
- C. Force (N)

D-F. Commercial cotton sock material testing

- E. Sock length (mm)
- F. Force (N)

G-I. Commercial diabetic sock materials testing

- H. Sock length (mm)
- I. Force (N)

J-L. Foot-stiffening sock materials testing

- K. Sock length (mm)
- L. Force (N)

M-N. Slack lengths of all tested socks

Tab: Statistical Data

A. Participant (number)
B. Condition (nylon or foot-stiffening sock)
C. Metabolic power (W/kg)
D. Stride time (s)
E. Contact time (s)
F. Stance length (m)
G. Duty factor
I. Peak vertical GRF (*BW)
K. Peak ankle moment (Nm/kg)
L. Average ankle moment (Nm/kg)
M. Peak ankle power (W/kg)
N. Ankle positive work (J/kg)
O. Peak ankle angular velocity (deg/s)
P. Average plantar flexion velocity (deg/s)
Q. Peak gear ratio
R. Average gear ratio
S. Ankle angle at toe-off (deg)
T. Peak MTPJ angle (deg)
V. Integrated TA rate (%MVC/s)
W. Integrated MG rate (%MVC/s)
X. Integrated SOL rate (%MVC/s)
Z. Average SOL fascicle velocity (mm/s)

AA. Average SOL fascicle length (mm)

AB. Average SOL pennation angle (deg)

AC. SOL velocity at peak (mm/s)

AD. SOL_length at peak (mm)

AE. SOL pennation angle at peak force (deg)

AF. Peak SOL muscle force (Nm/kg)

AG. Average SOL muscle force (Nm/kg)

AH. Average active SOL muscle volume (cm^3/kg)

AI. Average active SOL muscle volume rate (cm^3/kg/s)

AJ. Peak SOL muscle volume (cm^3/kg)

AK. Average SOL power (W/kg)

AL. Peak SOL fascicle power (W/kg)

AM. SOL mechanical work (J/kg)

AO. Average MG fascicle velocity (mm/s)

AP. Average MG fascicle length (mm)

AQ. Average MG pennation angle (deg)

AR. MG velocity at peak (mm/s)

AS. MG length at peak (mm)

AT. MG pennation at peak (deg)

AU. Peak MG muscle force (Nm/kg)

AV. Average MG muscle force (Nm/kg)

AW. Peak MG fascicle power (W/kg)

AX. Average MG fascicle power (W/kg)

AY. MG fascicle work (J/kg)

AZ. Average active MG muscle volume method 1 (cm^3/kg)

BA. Average active MG muscle volume rate method 1 (cm^3/kg/s)

BB peak MG muscle volume method 1 (cm^3/kg)

BD. Average MG+SOL active muscle volume method 1 (cm^3/kg)

BE. Average MG+SOL active muscle volume rate method 1 (cm^3/kg/s)

BF. Peak MG+SOL muscle volume method 1 (cm^3/kg)

BH. Average MG muscle volume rate method 2 (cm^3/kg/s)

BI. Average SOL active muscle volume rate method 2 (cm^3/kg/s)

BJ. Average MG+SOL active muscle volume rate method 2 (cm^3/kg/s)

Tab: Foot mechanics

A. Participant (number)
B. Condition (nylon or foot-stiffening sock)
C. Forefoot width (cm)
D. Forefoot circumference (cm)
E. Tensile force (N)
F. Forefoot pressure (mmHg)
G. Arch standing height (mm)
H. MLA arch stiffness (N/m)
I. Forefoot splay (cm)

Tab: Ensembled curves

A. Normalized stance phase

B-E. Vertical ground reaction force (*BW)

- B. Nylon condition’s mean
- C. Nylon condition’s standard error
- D. Foot-stiffening sock condition’s mean
- E. Foot-stiffening sock condition’s standard error

G-J. Ankle moment (Nm/kg)

- G. Nylon condition’s mean
- H. Nylon condition’s standard error
- I. Foot-stiffening sock condition’s mean
- J. Foot-stiffening sock condition’s standard error

L-O. Ankle angular velocity (deg/s)

- L. Nylon condition’s mean
- M. Nylon condition’s standard error
- N. Foot-stiffening sock condition’s mean
- O. Foot-stiffening sock condition’s standard error

Q-T. Ankle power (W/kg)

- Q. Nylon condition’s mean
- R. Nylon condition’s standard error
- S. Foot-stiffening sock condition’s mean
- T. Foot-stiffening sock condition’s standard error

V-Y. Gear ratio (r/R)

- V. Nylon condition’s mean
- W. Nylon condition’s standard error
- X. Foot-stiffening sock condition’s mean
- Y. Foot-stiffening sock condition’s standard error

AA-AD. MG L/L0

- AA. Nylon condition’s mean
- AB. Nylon condition’s standard error
- AC. Foot-stiffening sock condition’s mean
- AD. Foot-stiffening sock condition’s standard error

AF-AI. MG V/Vmax

- AF. Nylon condition’s mean
- AG. Nylon condition’s standard error
- AH. Foot-stiffening sock condition’s mean
- AI. Foot-stiffening sock condition’s standard error

AK-AN. SOL L/L0

- AK. Nylon condition’s mean
- AL. Nylon condition’s standard error
- AM. Foot-stiffening sock condition’s mean
- AN. Foot-stiffening sock condition’s standard error

AP-AS. SOL V/Vmax

- AP. Nylon condition’s mean
- AQ. Nylon condition’s standard error
- AR. Foot-stiffening sock condition’s mean
- AS. Foot-stiffening sock condition’s standard error

AU-AX. MG muscle force (N/kg)

- AU. Nylon condition’s mean
- AV. Nylon condition’s standard error
- AW. Foot-stiffening sock condition’s mean
- AX. Foot-stiffening sock condition’s standard error

AZ-BC. SOL muscle force (N/kg)

- AZ. Nylon condition’s mean
- BA. Nylon condition’s standard error
- BB. Foot-stiffening sock condition’s mean
- BC. Foot-stiffening sock condition’s standard error

BE-BH. MG pennation angle (deg)

- BE. Nylon condition’s mean
- BF. Nylon condition’s standard error
- BG. Foot-stiffening sock condition’s mean
- BH. Foot-stiffening sock condition’s standard error

BJ-BM. SOL pennation angle (deg)

- BJ. Nylon condition’s mean
- BK. Nylon condition’s standard error
- BL. Foot-stiffening sock condition’s mean
- BM. Foot-stiffening sock condition’s standard error

BO-BR. SOL active muscle volume method 1 (cm^3/kg)

- BO. Nylon condition’s mean
- BP. Nylon condition’s standard error
- BQ. Foot-stiffening sock condition’s mean
- BR. Foot-stiffening sock condition’s standard error

BT-BW. MG active muscle volume method 1 (cm^3/kg)

- BT. Nylon condition’s mean
- BU. Nylon condition’s standard error
- BV. Foot-stiffening sock condition’s mean
- BW. Foot-stiffening sock condition’s standard error

BY-CB. SOL+MG active muscle volume method 1(cm^3/kg)

- BY. Nylon condition’s mean
- BZ. Nylon condition’s standard error
- CA. Foot-stiffening sock condition’s mean
- CB. Foot-stiffening sock condition’s standard error

CD-CG. SOL RMS (%MVC)

- CD. Nylon condition’s mean
- CE. Nylon condition’s standard error
- CF. Foot-stiffening sock condition’s mean
- CG. Foot-stiffening sock condition’s standard error

CI-CL.	MG RMS (%MVC)

- CI. Nylon condition’s mean
- CJ. Nylon condition’s standard error
- CK. Foot-stiffening sock condition’s mean
- CL. Foot-stiffening sock condition’s standard error

CN-CQ. TA RMS (%MVC)

- CN. Nylon condition’s mean
- CO. Nylon condition’s standard error
- CP. Foot-stiffening sock condition’s mean
- CQ. Foot-stiffening sock condition’s standard error

CS-CV. MTPJ angle (deg)

- CS. Nylon condition’s mean
- CT. Nylon condition’s standard error
- CU. Foot-stiffening sock condition’s mean
- CV. Foot-stiffening sock condition’s standard error

CX-DA. Ankle angle (deg)

- CX. Nylon condition’s mean
- CY. Nylon condition’s standard error
- CZ. Foot-stiffening sock condition’s mean
- DA. Foot-stiffening sock condition’s standard error

Tab: Hip and knee joint mechanics

A. Normalized stance phase

B-E. Hip joint angles (deg)

- B. Nylon condition’s mean
- C. Nylon condition’s standard error
- D. Foot-stiffening sock condition’s mean
- E. Foot-stiffening sock condition’s standard error

G-J. Hip joint moment (Nm/kg)

- G. Nylon condition’s mean
- H. Nylon condition’s standard error
- I. Foot-stiffening sock condition’s mean
- J. Foot-stiffening sock condition’s standard error

L-O. Hip joint power (W/kg)

- L. Nylon condition’s mean
- M. Nylon condition’s standard error
- N. Foot-stiffening sock condition’s mean
- O. Foot-stiffening sock condition’s standard error

Q-T. Knee joint angle (deg)

- Q. Nylon condition’s mean
- R. Nylon condition’s standard error
- S. Foot-stiffening sock condition’s mean
- T. Foot-stiffening sock condition’s standard error

V-Y. Knee joint moment (Nm/kg)

- V. Nylon condition’s mean
- W. Nylon condition’s standard error
- X. Foot-stiffening sock condition’s mean
- Y. Foot-stiffening sock condition’s standard error

AA-AD. Knee joint power (W/kg)

- AA. Nylon condition’s mean
- AB. Nylon condition’s standard error
- AC. Foot-stiffening sock condition’s mean
- AD. Foot-stiffening sock condition’s standard error
