# Supplementary material for: Foot-stiffening socks reduce the metabolic cost of walking
Source: Proc Biol Sci. 2026 Nov 4;293(2082):20261467. doi: 10.1098/rspb.2026.1467 (PMC13730810; doi:10.1098/rspb.2026.1467)
Supplement: Supplementary file 1 [file rspb.2026.1467.zip › rspb.2026.1467/rspb20261467_si_003.docx]

Supplementary Figures

**Figure S1.** **Foot-stiffening socks did not affect metatarsophalangeal and ankle joint angle.** (Left) Ensembled profiles of metatarsophalangeal (MTP) and ankle joint angle during the stance-phase of walking. (Right) Avg ± SE (circle symbols) and individual participant data (square symbols) during walking in control socks (orange) and foot-stiffening socks (purple). There were no statistical differences between average hip joint angle, moment and power in compliant control socks and foot-stiffening socks (p > 𝛼).

**Figure S2.** **Foot-stiffening socks did not affect hip joint mechanics.** (Left) Ensembled profiles of hip joint angle, moment, and mechanical power during the stance-phase of walking. (Right) Avg ± SE (circle symbols) and individual participant data (square symbols) during walking in control socks (orange) and foot-stiffening socks (purple). There were no statistical differences between average hip joint angle, moment and power in compliant control socks and foot-stiffening socks (p > 𝛼).

**Figure S3.** **Foot-stiffening socks did not affect knee joint mechanics.** (Left) Ensembled profiles of knee joint angle, moment, and mechanical power during the stance-phase of walking. (Right) Avg ± SE (circle symbols) and individual participant data (square symbols) during walking in control socks (orange) and foot-stiffening socks (purple). There were no statistical differences between average knee joint angle, moment and power in compliant control socks and foot-stiffening socks (p > 𝛼).

**Figure S4.** **Foot-stiffening socks reduced medial gastrocnemius shortening velocity.** The ensembled curves of medial gastrocnemius fascicle mechnics during walking’s stance phase for control socks (orange) and foot-stiffening socks (purple). Fascicle (A) forces, (B) pennation angles, (C) length with respect to the optimal length for active force production (L0), and (D) velocity with respect to the maximal shortening velocity (Vmax). The presented curves were medial gastrocnemius fascicle Avg (± SE) data. Foot-stiffening socks decreased average medial gastrocnemius shortening velocity (d = 0.52, p = 0.034). There were no statistical differences between average fascicle force, pennation angle, or operating length in compliant control socks and foot-stiffening socks (p > 𝛼).

**Figure S5.** **Medial gastrocnemius (MG) and soleus (SOL) active muscle volume during walking.** (A) medial gastrocnemius and (B) soleus Avg (± SE) active muscle volume data during the stance-phase of walking in control socks (orange) and foot-stiffening socks (purple). Foot-stiffening socks reduced the average active muscle volume of medial gastrocnemius (d = 0.25, p = 0.029) and soleus (d = 0.50, p = 0.003) muscles.

**Figure S6.** **Automated Sock-Knitting Machine that customized the foot-stiffening socks.** Using an automated sock-knitting machine (LEIERFU, Zhejiang, China), we programmed the desired sock width, length, thickness, and material composition to knit the foot-stiffening socks.

**Figure S7.** **Foot-stiffening socks did not affect leg muscle activation.** (A) Tibialis anterior, (B) medial gastrocnemius, and (C) soleus root mean squared (RMS) electromyography signal during the stance-phase of walking in control socks (orange) and foot-stiffening socks (purple) scaled to the respective muscle’s maximum voluntary contraction (MVC).
